# Exploring the Role of Diabetes in ALS: A Population-Based Cohort Study

**DOI:** 10.3390/life15060936

**Published:** 2025-06-10

**Authors:** Ilaria Martinelli, Giulia Gianferrari, Rebecca Santarelli, Elisabetta Zucchi, Cecilia Simonini, Nicola Fini, Andrea Ghezzi, Annalisa Gessani, Laura Ferri, Krzysztof Smolik, Diana Ferraro, Roberta Bedin, Matteo Gizzi, Elisabetta Sette, Veria Vacchiano, Luigi Bonan, Lucia Zinno, Patrizia De Massis, Elena Canali, Doriana Medici, Emilio Terlizzi, Simonetta Morresi, Mario Santangelo, Alberto Patuelli, Marco Currò Dossi, Marco Longoni, Maura Pugliatti, Tommaso Filippini, Salvatore Ferro, ERRALS Study Group, Jessica Mandrioli

**Affiliations:** 1 Department of Neurosciences, Azienda Ospedaliero Universitaria di Modena, 41126 Modena, Italy; martinelli.ilaria@aou.mo.it (I.M.); zucchi.elisabetta@aou.mo.it (E.Z.); ceciliasimonini24@gmail.com (C.S.); fini.nicola@aou.mo.it (N.F.); gessani.annalisa@aou.mo.it (A.G.); 98506@studenti.unimore.it (K.S.); ferraro.diana@aou.mo.it (D.F.); roberta.bedin@unimore.it (R.B.); jessica.mandrioli@unimore.it (J.M.); 2 Neuroscience Ph.D. Program, University of Modena and Reggio Emilia, 41125 Modena, Italy; ghezzi.andrea@aou.mo.it (A.G.); ferrilaura@outlook.it (L.F.); 3 Department of Biomedical, Metabolic and Neural Sciences, University of Modena and Reggio Emilia, 41125 Modena, Italy; 253886@studenti.unimore.it (R.S.);; 4 Department of Neurology, IRCCS Arcispedale Santa Maria Nuova, 42123 Reggio Emilia, Italy; elena.canali@asmn.re.it; 5 Department of Neurology, Faenza and Ravenna Hospital, 48100 Ravenna, Italy; matteo.gizzi@auslromagna.it (M.G.); marco.currodossi@auslromagna.it (M.C.D.); 6 Department of Neurology, St. Anna Hospital, 44124 Ferrara, Italy; e.sette@ospfe.it; 7 IRCCS Istituto delle Scienze Neurologiche di Bologna, 40139 Bologna, Italy; veria.vacchiano2@unibo.it (V.V.); luigi.bonan@studio.unibo.it (L.B.); 8 Department of General and Specialized Medicine, University Hospital of Parma, 43126 Parma, Italy; lzinno@ao.pr.it; 9 Department of Neurology, Imola Hospital, 40026 Bologna, Italy; p.demassis@ausl.imola.bo.it; 10 Department of Neurology, Fidenza Hospital, 43036 Parma, Italy; dmedici@ausl.pr.it; 11 Department of Neurology, G. Da Saliceto Hospital, 29121 Piacenza, Italy; e.terlizzi@ausl.pc.it; 12 Department of Neurology, Bufalini Hospital, 47521 Cesena, Italy; simonetta.morresi@auslromagna.it (S.M.); marco.longoni@auslromagna.it (M.L.); 13 Department of Neurology, Carpi Hospital, 41012 Modena, Italy; m.santangelo@ausl.mo.it; 14 Department of Neurology, Forlì Hospital, 47121 Forlì, Italy; alberto.patuelli@auslromagna.it; 15 Department of Neurosciences and Rehabilitation, University of Ferrara, 44121 Ferrara, Italy; pglmra@unife.it; 16 Research Centre in Environmental, Genetic and Nutritional Epidemiology—CREAGEN, University of Modena and Reggio Emilia, 41125 Modena, Italy; 17 School of Public Health, University of California Berkeley, Berkeley, CA 94704, USA; 18 Department of Hospital Services, Emilia Romagna Regional Health Authority, 40127 Bologna, Italy; salvatore.ferro@regione.emilia-romagna.it

**Keywords:** amyotrophic lateral sclerosis, diabetes mellitus, disease progression, metabolism, tracheostomy, respiratory dysfunction

## Abstract

Type 2 diabetes mellitus (T2DM) as a comorbidity in amyotrophic lateral sclerosis (ALS) has sparked interest for its potential impact on disease expression and prognosis. In this retrospective cohort study, we investigated the prevalence and clinical correlates of T2DM in a large cohort of patients from the ALS registry of a Northern Italy region, Emilia Romagna, established in 2009. Out of 1756 ALS patients enrolled up to 2021, 145 were affected by T2DM (diALS). Patients with diALS were older than those without T2DM (ndALS) (71.56 vs. 65.76 years, *p* < 0.001), had a higher body mass index (25.63 vs. 24.23, *p* < 0.001), but experienced greater weight loss at diagnosis (6.87% vs. 5.44%, *p* < 0.007). Respiratory onset (6.2% vs. 2.6%, *p* = 0.013) and respiratory phenotype (4.2% vs. 1.4%, *p* = 0.04) were more frequent among diALS. Coherently, diALS presented a lower forced vital capacity (74.9% vs. 87.9%, *p* ≤ 0.001) and more frequently adopted Non-Invasive Ventilation (NIV) (50.35% vs. 37.61%, *p* = 0.003), with significant influence on time to NIV (HR 1.71, 95% CI 1.07–2.74, *p* = 0.024). Exploring genetic background, among all the genes examined *C9ORF72* emerged as underrepresented among diALS (7.64% in ndALS vs. 0% in diALS, *p* = 0.039). In conclusion, we confirmed a more severe respiratory dysfunction in diALS, suggesting a specific frailty in respiratory muscles, together with some peculiar clinical features consistent with the previous literature data, such as a later onset. The lower prevalence of *C9ORF72* expansion in this population may hint towards a specific role of the gene in metabolism and inflammation, granting more space to non-genetic causes, warranting further studies for confirmation.

## 1. Introduction

Amyotrophic lateral sclerosis (ALS) is a rare neurodegenerative disorder characterized by the progressive deterioration of motoneurons localized in the primary motor cortex, corticospinal tracts, brainstem, and spinal cord. The most common form of ALS involves the degeneration of both upper and lower motor neurons, leading to a gradual decline in muscle function and death from respiratory failure within 2–5 years [1]. Several phenotypes of ALS have been described, showing significant differences in progression, prognosis, and symptom distribution. These range from the bulbar or classic phenotype, characterized by both upper and lower motor neuron involvement—albeit with different distributions—and a median survival of 2 to 3 years, to the Upper Motor Neuron predominant (UMN-p) phenotype and flail leg and flail arm phenotypes, which are marked by a predominance of upper or lower motor neuron signs, respectively, and are associated with a longer survival [2,3]. Type 2 Diabetes Mellitus (T2DM) arises from increased resistance to insulin action [4]. According to the International Diabetes Federation, diabetes was responsible for 4.2 million deaths and affected 463 million adults aged 20–79 years in 2019. Projections estimate that this number will rise to 700 million by 2045 [5]. Patients with T2DM have a 15% higher mortality rate compared to non-diabetic individuals and face a significantly increased risk of developing ischemic stroke, coronary heart disease, and other vascular complications [6]. 

Neurological complications of T2DM include distal symmetric polyneuropathy, autonomic neuropathy, and focal or multifocal neuropathies (e.g., cranial nerve palsies, carpal tunnel syndrome), as well as diabetic amyotrophy and diabetic myopathy affecting the peripheral nervous system [7]. Additionally, diabetic encephalopathy, stroke and cerebrovascular disease, and hypoglycemia-related neurological symptoms involve the central nervous system [8]. Several studies have explored the relationship between T2DM and ALS yielding conflicting results. The prevailing hypothesis suggests that T2DM may exert a protective effect against the risk of developing ALS [9], possibly through indirect or direct mechanisms. For instance, T2DM is commonly associated with dyslipidemia, a factor that has been associated with better prognosis in ALS patients [10,11]. Additionally, a high body mass index (BMI), often observed in diabetic individuals, has been demonstrated as protective in ALS across various studies [12,13,14,15]. Notably, while T2DM may have protective associations, type 1 diabetes is linked to factors that worsen ALS prognosis, such as lower BMI [16,17]. Moreover, while some studies suggest that individuals with T2DM may develop ALS at a later age and experience slower disease progression [18], other research points to a more aggressive disease course, characterized by early respiratory involvement [19].

The potential role of antidiabetic drugs as protective factors in ALS has also been explored [20]. For instance, pioglitazone and metformin may confer neuroprotection through antioxidant and anti-inflammatory properties—counteracting key processes implicated in ALS pathogenesis [21,22]. Additionally, T2DM might help mitigate the hypermetabolism commonly observed in ALS by enabling alternative energy production pathways, with hyperglycemia possibly exerting a protective effect [23,24]. Elevated levels of uric acid, frequently found in individuals with T2DM [25], have also been associated with a reduced risk of neurodegenerative diseases, including ALS [26,27,28]. However, it remains unclear whether T2DM itself has a protective role or if the observed effects are mediated by the combined influence of the aforementioned factors [23]. 

The aim of this study is to evaluate if T2DM may impact on clinical and phenotypic features, in particular on disease progression, prognosis, and survival rates, among ALS patients from the population-based registry of the Emilia-Romagna Region (ERRALS).

## 2. Methods

### 2.1. Study Population

In this retrospective cohort study we included all patients diagnosed with definite, clinically probable, probable with laboratory support, or possible ALS according to the Revised El Escorial Criteria (EEC-R) [29] between 1 January 2009 and 1 June 2021, as recorded in ERRALS registry. All patients were followed up to 1 June 2023, or earlier in case of death or emigration from the region. After the first visit, we recorded follow-up visits approximately every three months or based on patients need, according to clinical practice for ALS [30]. Patients were treated in the frame of regular ALS multidisciplinary clinical practice, and therapeutic decisions and medical care during the treatment were carried out by the treating physicians and were not defined by a specific protocol. The presence of diabetes was ascertained based on plasma glucose criteria, either the fasting plasma glucose value or the 2-h plasma glucose value during a 75 g oral glucose tolerance test (OGTT), or A1C criteria [31]. In our registry, there were no cases of ALS patients with a diagnosis of T1DM. ALS patients with a history of T2DM at diagnosis (diALS) were compared to those without T2DM at diagnosis (ndALS). 

### 2.2. Clinical Measures 

Demographic data collected for this study included sex, date and place of residence, and date of birth. Clinical information included the date of disease onset, date of diagnosis, site of onset, and phenotype categorized as classic, bulbar, predominant upper motor neuron (UMN), flail arm or flail leg, respiratory [32]. Cognitive impairment was recorded, specifying its presence and type according to the Strong criteria [33]. Data on height, body weight, and BMI were also collected. Family history of ALS, frontotemporal dementia (FTD), Parkinson’s disease (PD), and Alzheimer’s disease (AD) was documented. The *C9ORF72* repeat expansion status was assessed using a repeat-primed PCR method with laboratory-specific validation and quality control performed through Southern blot analysis. Genetic analyses systematically included sequencing of *SOD1*, *FUS*, *TARDBP*. Based on clinical presentation and historical context, a subset of patients underwent a next-generation sequencing (NGS) approach using a customized probe-based panel (Illumina Nextera Rapid Capture Custom kit, Illumina, San Diego, CA, USA), which targets 78 genes [34]. 

In addition to T2DM, comorbidities were recorded and categorized into neurodegenerative diseases; cardiovascular conditions, such as hypertension and heart disease; thyroid dysfunction; metabolic disorders; chronic obstructive pulmonary disease (COPD) and other respiratory conditions; gastrointestinal, urological, hematological, autoimmune, neoplastic diseases; and psychiatric disorders [35]. Neurological examinations were documented at the first visit and at each follow-up, alongside measurements of body weight and BMI. Data on weight prior to symptom onset were collected, and weight loss at diagnosis was analyzed both as a categorical variable (≥1 kg loss between pre-onset and diagnosis), and as a quantitative variable (absolute and percentage weight loss). Respiratory function was assessed through spirometry with forced vital capacity (FVC) at diagnosis and during follow-ups as per clinical practice [34]. The ALS Functional Rating Scale-Revised (ALSFRS-R) was evaluated at each visit. Disease progression at diagnosis was quantified as the monthly decline in ALSFRS-R scores from the maximum score of 48 points at disease onset [36]. Data on the use of nutritional support, Percutaneous Endoscopic Gastrostomy (PEG), and Non-Invasive or Invasive Ventilation (NIV or IV) were recorded. Missing data were retrieved and verified through administrative records [37]. Finally, patient outcomes, including the date and cause of death, were documented.

### 2.3. Statistics

Descriptive statistics were performed using Student’s *t*-test or ANOVA and Chi-squared test when appropriate. Linear regression and logistic regression were applied to study the association between dependent and independent variables. Correlations were studied with Pearson’s test. Survival was calculated from onset to death/tracheostomy or the censoring date (last follow-up, 1 June 2023) using the Kaplan–Meier method; log-rank test was used for group comparison. Univariate Cox regression analysis was employed to validate the prognostic impact of possible covariates. Multivariable analysis using stepwise backward method with the Breslow method for ties, incorporating variables with a *p*-value < 0.1 from the univariate analysis was then applied to study the role of independent variables on survival. Missing data were not substituted, and they were treated as such. Analyses were performed with STATA version 18 (StataCorp LLC, College Station, TX, USA, 2023). 

## 3. Results

### 3.1. Clinical and Demographic Features

This study included 1756 patients diagnosed with ALS in the Emilia-Romagna Region since 1 January 2009. Among these, we identified 145 (8.26%) patients as having T2DM (diALS), while 1611 patients had no history of T2DM (ndALS). The clinical and demographic characteristics of the study population are summarized in Table 1. A more pronounced male predominance was observed among diALS patients compared to ndALS (66.21% vs. 54.50%, *p* = 0.007). Patients with diALS were older at ALS onset compared to ndALS (72.89 years in diALS and 66.82 years in ndALS, *p* < 0.001), but presented with a lower ALSFRS-R score at diagnosis (36.70 ± 7.63 vs. 38.85 ± 7.17, *p* = 0.001), and a higher BMI at diagnosis (25.63 ± 4.63 vs. 24.23 ± 3.89, *p* < 0.001). Respiratory onset was significantly more frequent in the diALS group compared to the ndALS group (6.21% vs. 2.61%, *p* = 0.013), while distal upper limb onset was significantly less frequent in diALS patients compared to ndALS patients (16.55% vs. 26.57%, *p* = 0.008).

Regarding family history of neurodegenerative diseases (including ALS, dementia, and Parkinson’s disease), diALS patients showed a lower frequency compared to ndALS (for ALS 5.71% in ndALS vs. 2.07% in diALS, *p* = 0.063; for dementia 11.61% in ndALS vs. 6.90% in diALS, *p* = 0.085, for Parkinson’s disease 4.66% in ndALS vs. 5.52 in diALS, *p* = 0.640) (Table S1). When considering the EEC-R, none of the four classes (definite, clinically probable, probable laboratory supported, possible) were distributed differently across the two groups, as shown in Table S2.

Symptoms at onset according to diabetic status, specifically fasciculations, cramps, motor deficit, and spasticity, are reported in Table 2. Onset with motor impairment was considerably more frequent among diALS (95.17% vs. 58.59%, *p* = 0.007), whereas spasticity was more common among ndALS (6.33% vs. 2.07%, *p* = 0.038). 

### 3.2. Genetics and Phenotypes

Among diALS patients, the most frequently observed mutation was in *SOD1* (3.92%) followed by *FUS* (2%), whereas in the ndALS group, the most common mutations were in *C9ORF72* (7.64%) and *SOD1* (3.85%) genes. Interestingly, for diALS we observed a poor representation of mutations in the remaining causative and other susceptibility genes (Table 3a). *C9ORF72* expansion was absent in the diALS subgroup (7.64% in ndALS vs. 0% in diALS, *p* = 0.039). Regarding phenotype, the respiratory phenotype was more frequently associated with the presence of T2DM in our cohort (1.45% in ndALS vs. 4.24% in diALS, *p* = 0.045) (Table 3b).

Since genetics and phenotype are related to age [38], we examined genotype, onset and respiratory features, and disease progression rate by age classes (Table 4).

Then we considered genotype, onset and respiratory features, and disease progression rate as dependent variables in regression models considering T2DM and age as independent variables. The respiratory onset was significantly associated with T2DM (OR = 2.21, 95% CI: 1.04–4.69, *p* = 0.039), after adjusting for age using logistic regression. Logistic regression analysis revealed that older age at onset was associated with a lower probability of carrying the *C9ORF72* expansion, with an OR = 0.57, 95% CI: 0.42–0.79, *p* = 0.001. Quantile regression analysis revealed that T2DM was significantly associated with progression rate (β = 0.31, 95% CI: 0.16 to 0.46, *p* < 0.001), after adjusting for age. T2DM was also significantly associated with a low FVC (β = −14.33, 95% CI: −22.04 to −6.61, *p* < 0.001), after adjusting for age. 

### 3.3. Comorbidities and Interventions

In our study, diALS patients were more frequently affected by Parkinson’s disease (4.83% vs. 1.80%, *p* = 0.014) and vascular dementia (0.69% vs. 0.6%, *p* = 0.032), as detailed in Table 5. Concerning concomitant respiratory diseases, only COPD was more prevalent among diALS patients (12.41% vs. 7.20%, *p* = 0.024). As expected, cardiac comorbidities were differently distributed between the two groups. Specifically, among diALS patients, ischemic cardiopathy was the most common (15.17%, followed by heart conduction disorders (8.27%), which included atrial fibrillation and atrioventricular blocks.

### 3.4. Impact of Diabetes, Comorbidities, and Clinical Features on Disease Progression

Since T2DM was associated with several comorbidities, possibly impacting on disease progression, we tested the impact of diabetes, clinical features, comorbidities, and drugs on progression rate measured at diagnosis using multivariable regression (Table 6). The presence of T2DM had a negative impact on progression rate at diagnosis, (β = 0.45, 95%CI 0.14–0.75, *p* = 0.004). Additionally, progression was influenced by weight loss at diagnosis (β = 0.03, 95% CI 0.02–0.04, *p* < 0.001) and the presence of the C9ORF72 expansion (β = 0.42, 95% CI 0.12–0.73, *p* = 0.006).

### 3.5. Pharmacological and Non-Pharmacological Treatments for ALS

Table S3 provides a comprehensive overview of medications taken by the patients under review. Among these, the only medication significantly differing between patients with and without T2DM was Acetyl-L-Carnitine, taken by 28.93% of ndALS patients compared to 17.24% of diALS patients (*p* = 0.003). NIV was more frequently used by diALS patients (50.35%) compared to ndALS patients (37.61%, *p* = 0.03), as shown in Table S4. Since NIV usage can be influenced by multiple factors beyond T2DM, we performed a logistic regression analysis examining the association between T2DM and NIV usage as the dependent variable, adjusting for other independent variables. The results indicated an increased risk for NIV treatment in patients with T2DM (OR 1.72, 95% CI 0.98–3.02, *p* = 0.057), with a respiratory onset (OR 5.1, 95%CI 1.67–15.58, *p* = 0.004) and with a higher BMI at diagnosis (OR 1.04, 95% CI 1.0–1.08, *p* = 0.041), while a reduced risk of NIV was reported for patients with lower FVC (OR 0.99, 95% CI 0.98–1.00, *p* = 0.007) and older age at onset (OR 0.98, 95% CI 0.97–1.00), as shown in Table 7.

### 3.6. Tracheostomy-Free Survival Analysis

**Figure 1:**
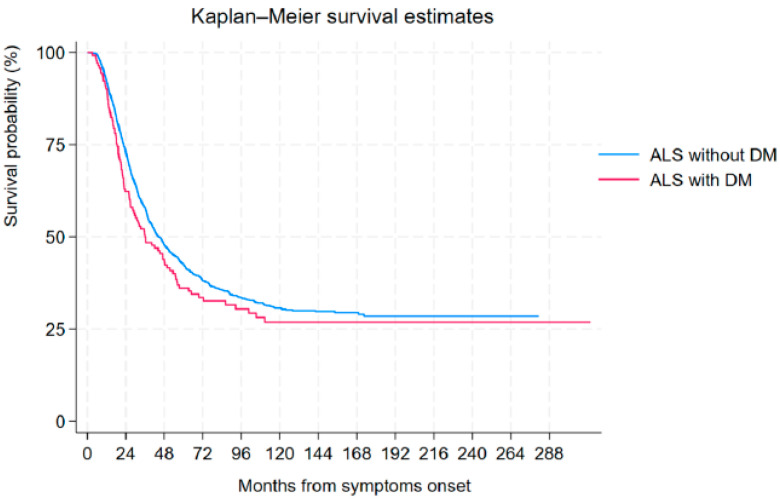
Kaplan–Meier survival estimates for ALS with and without T2DM.

The median tracheostomy-free survival from symptom onset in the entire ALS patient cohort was 43.81 months (95% CI: 40.52–47.95 months). When considering the presence of T2DM, ndALS presented a median survival of 44.43 months (95% CI: 41.01-–48.51 months), whereas diALS had a median survival of 35.89 months (95% CI: 26.95–49.66 months) (*p* = 0.138) (Figure 1).

Univariate Cox regression analysis identified several significant factors associated with overall survival for the general population, including certain comorbidities, though diabetes itself was not statistically significant (see Table S5). Subsequently, a multivariable analysis was performed using the Cox regression model with the Breslow method for ties, incorporating variables with a *p*-value < 0.1 from the univariate analysis to determine independent prognostic factors for survival. T2DM did not result as an independent prognostic factor for survival (HR 1.09, 95% CI 0.85–1.40, *p* = 0.474). The results, detailed in Table 8, demonstrated that independent prognostic factors associated with a better survival include a diagnosis of “Possible” ALS according to Revised El Escorial Criteria (EEC-R), longer diagnostic delay, and higher ALSFRS-R scores at diagnosis. Conversely, older age at onset, a “Definite” ALS according to EEC-R, the presence of FTD, and faster progression rates emerged as negative independent prognostic factors for survival. 

The same survival analysis among ndALS showed that the factors independently associated with a worse survival at multivariable analysis were a short diagnostic delay, a higher age at onset, bulbar onset, weight loss, progression rate, and the presence of FTD (Table S6).

Focusing on ALS patients affected by diabetes, the variables that were identified as significant factors impacting survival at univariate analysis are detailed in Table S7. The multivariable analysis with the above-mentioned parameters, revealed as independent factors associated with a worse prognosis the following parameters: a short diagnostic delay, a “Definite” ALS diagnosis according to EEC-R, a lower BMI, progression rate ad diagnosis, concomitant FTD, and respiratory onset, as detailed in Table 9.

The combination of T2DM and hypertension in ALS patients was associated with a median survival of 32.40 months, compared to 69.41 months in non-hypertensive individuals (*p* = 0.083). Furthermore, significant reductions in median survival were observed in diALS patients with weight loss (26.95 vs. 42 months) and those with FTD (18.67 vs. 40.10 months) compared to ndALS patients. Among diALS patients, those with a respiratory onset had a markedly shorter median tracheostomy-free survival (13.7 months) compared to those with other onset types (42 months).

### 3.7. Influence of Diabetes on Respiratory Function

#### 3.7.1. Diabetes and Other Clinical Variables and Non-Invasive Ventilation

The variables associated with time to NIV in patients who received NIV in the univariate analysis are detailed in Table S8. After conducting multivariable analysis on these data, T2DM acts as an independent factor influencing negatively time to NIV (HR 1.71, 95% CI 1.07–2.74, *p* = 0.024). Interestingly, respiratory onset, hypertension and cardiovascular diseases, weight loss, and progression rate persist as negative variables on time to NIV, while diagnostic delay, and phenotypes different from bulbar were found to have a positive influence (Table 10). 

#### 3.7.2. Impact of Diabetes and Other Clinical Variables on Invasive Ventilation

Several factors were significantly associated with time to IV in those who received IV at univariate analysis, as represented in Table S9. The results of the multivariable Cox regression analysis revealed that T2DM was not associated with IV(HR 0.95, 95% CI 0.47–1.93, *p* = 0.897). Variables with positive impact on time to IV include diagnostic delay and phenotype different from bulbar, while age at onset, progression rate, cardiovascular diseases and *C9ORF72* expansion act negatively on time to IV (Table 11).

## 4. Discussion

The objective of this study was to assess the differences in phenotypic and genotypic characteristics, and in disease progression, prognosis, and survival rates, among ALS patients with and without T2DM included in the ERRALS registry. The most relevant finding from our study concerns respiratory frailty in diALS subgroup. Specifically, respiratory onset and low FVC were significantly associated with T2DM, even after adjusting for age using logistic regression. Consistently, when analyzing clinical phenotypes, we observed that the respiratory phenotype was more prevalent in diALS compared to ndALS patients. Furthermore, diALS patients were more likely to receive NIV than their ndALS counterparts, in line with recent studies suggesting that T2DM may impair the phrenic nerve and accelerate respiratory decline [19]. In our population, a higher percentage of diALS patients utilized NIV, and T2DM was significantly associated with time to NIV, while T2DM did not emerge as an independent prognostic factor influencing time to IV, which instead were influenced by other well-known clinical and demographic factors such as diagnostic delay [37,39,40,41], concurrent cardiovascular diseases [35], disease progression rate [41], *C9ORF72* expansion [42], and phenotype [2,35,43]. McDonald et al. demonstrated that glucose metabolism is disrupted in ALS models, with impaired insulin signaling and altered glucose uptake in skeletal muscles, which could compromise muscle energy supply and function [44]. Additionally, a metabolic switch from glucose to lipid utilization in skeletal muscles has been observed early in ALS progression, which may exacerbate muscle weakness and denervation [45]. This metabolic dysregulation may extend to respiratory muscles, contributing to their increased frailty in diALS patients. This is consistent with the hypothesis that metabolic derangements in T2DM exacerbate ALS respiratory severity via mechanisms including mitochondrial impairment and chronic inflammation [46,47]. Therefore, the specific frailty of respiratory muscles in diALS may result from combined effects of metabolic alterations, mitochondrial dysfunction, and systemic inflammatory processes. These mechanisms warrant further investigation to clarify their roles and to identify potential therapeutic targets. While our data suggest T2DM accelerates respiratory decline in ALS, reverse causality (e.g., ALS progression causing weight loss and transient glycemic improvements) cannot be excluded. Unmeasured confounders, such as physical inactivity or antidiabetics drugs, may also contribute. Prospective studies with serial metabolic profiling are needed to clarify causality.

DiALS patients experienced disease onset later than the ndALS counterpart. Although some population-based studies have documented no difference in the age of onset between ALS patients based on T2DM status [48], our findings align with the majority of studies that have observed a delayed onset—by approximately four years—in patients with T2DM or pre-existing diabetes [9,15,49]. The role of T2DM in delaying ALS onset remains uncertain. While it is well-established that individuals with multiple chronic conditions often experience worse outcomes [50], emerging evidence suggests that the biological mechanisms and pathways involved in T2DM at the cellular level may not only trigger but may also interact with those involved in ALS pathogenesis. For example, individuals with T2DM have been reported to exhibit elevated concentrations of progranulin [51], an adipokine involved in insulin resistance induced by high fat intake. Notably progranulin overexpression has been reported to counteract axonopathy induced by mutant TDP-43 in vivo [52]. In this context, it is plausible that higher serum levels of lipids or glucose might act as compensatory mechanisms in patients at risk of developing ALS, contributing to the delay of ALS onset [9].

DiALS also exhibited a lower total score on the ALSFRS-R and a lower FVC at diagnosis, suggesting a more aggressive disease course from onset. Consistently, the progression rate at diagnosis was higher in diALS patients, who also experienced marked weight loss despite a higher BMI at diagnosis. Regression analysis confirmed that the progression rate was independently influenced by T2DM, along with weight loss, suggesting a worse prognosis for diabetic patients, which appears to be related to diabetes itself rather than solely to associated demographic and clinical characteristics. The pathophysiological mechanisms connecting T2DM and ALS progression are still not fully understood due to limited evidence [53]. To disentangle the issue, an indication could come from the association between type 1 diabetes (characterized by an absolute lack of insulin and high blood sugar) and an increased ALS risk [12,54], which points to the possibility that T2DM’s protective effect on ALS might stem from unknown signaling pathways rather than from the currently proposed mechanisms. Recent observations have highlighted that the loss of nuclear TDP-43 contributes to the impaired early-phase insulin secretion seen in early-stage ALS patients [9,55,56] and that the depletion of nuclear TDP-43 in pancreatic β cells may play a role in the reduced insulin secretion in ALS patients [55].

Although diALS had higher progression rate at diagnosis, we confirmed that T2DM was not an independent factor influencing survival in ALS, according to literature data [35,48,57]. As far as survival is concerned, our data align with the study conducted by Paganoni et al. [58], which stated that diabetes did not influence the survival of ALS patients. An opposing view was put forth by Kioumourtzoglou et al., as in their study, diabetic patients exhibited lower survival compared to non-diabetics [12]. In our population, survival was influenced by established prognostic factors such as diagnostic delay, weight loss, and FTD, as previously reported [43,59]. The “definite” El Escorial classification was associated with worse survival also in the diabetic population, consistent with other studies [60,61]. Interestingly, BMI appeared to influence only diALS patients, suggesting a protective effect on survival. Additionally, ALSFRS-R scores and progression rates were significant factors impacting survival, in agreement with previous findings [34,62].

In our cohort, we examined the most common ALS-associated genetic mutations, and, among these, the *C9ORF72* expansion was found to be significantly less frequent in diALS patients compared to ndALS. Except for the *SOD1* and *FUS* genes, we observed a poor representation of mutations in other causative and other susceptibility genes among diabetic patients. This finding suggests that diALS cases are less likely to be driven by the genetic mechanisms often implicated in ALS pathogenesis. On the contrary, the absence of major genetic mutations in this subgroup may point toward alternative mechanisms, possibly metabolic or inflammatory in nature, that contribute to early or more pronounced respiratory involvement. In the literature, *C9ORF72* repeat expansions have been demonstrated to act in the differentially regulated splicing of several genes involved in cholesterol biosynthesis and glucose metabolism [63,64]. There are currently no studies specifically focused on the role of this mutated gene in diALS patients, as well as for the entire ALS population. Other possible explanations for the absence of *C9ORF72* in diALS may reflect sample size limitations and also could be that T2DM typically manifests predominantly after the age of 65 [65], while patients with *C9ORF72* expansion tend to experience earlier disease onset and reduced survival [12,66,67]. However, this does not account for the similar representation of other mutations, such as those in the *SOD1* gene, in both diALS and ndALS [68], nor does it explain the underrepresentation of mutations in other genes. Interestingly, some studies highlighted the potential for age-related differences in how DM impacts ALS development, showing that DM might even act protectively against ALS when diagnosed at a younger age, while becoming a risk factor if diagnosed later in life [18]. Furthermore, several other ’non-genetic’ factors—such as smoking, head injuries, and exposure to certain environmental toxins—have been linked to ALS. These factors tend to accumulate over time, potentially amplifying their impact in older individuals [69]. 

Vascular dementia and Parkinson’s disease were more frequent among diALS patients as well as COPD, heart diseases, and hypertension, as reported for the general population affected by T2DM [70,71]. Recently, T2DM has been described as an independent risk factor associated with more severe involvement in patients recently diagnosed with PD [72]. A possible link between PD and T2DM could be found in the insulin resistance, that can be targeted to ameliorate neurodegeneration [72]. One potential explanation for the effects of T2DM on ALS comorbidities may be a result of increased neurovascular burden. Arterial hypertension was an independent prognostic factor only for the diabetic population and may partially enhance the effects of T2DM on disease progression. Accordingly, several studies [35,73] suggested that hypertension was a risk factor for ALS. The study by Moreau et al. [73] on a population of 102 patients demonstrated that a 2-year presence of arterial hypertension could indeed influence and worsen prognosis by reducing neural perfusion and consequently damaging motor neurons [74]. In fact, there was a correlation between hypertension and respiratory pathologies such as obstructive sleep apnea syndrome, lung diseases, and hypoxemia [75]. 

The main strength of our study lies in our large retrospective cohort of ALS patients who were extensively phenotyped, allowing us to gain a global overview of ALS trajectory. Additionally, we had access to a substantial amount of relevant data on vascular risk factors. Nonetheless, there are some limitations in our study that require emphasis. Firstly, because of the retrospective observational nature of our study, it is difficult to assess the exact nature of the relationship between ALS and diabetes despite our attempts to adjust for some known confounding factors, such as hypertension, dyslipidemia, and cardiovascular disease. However, smoking history was not systematically recorded and could not be included. Additionally, data on lipid profiles and detailed glycemic control were not available, as well as biomarkers of respiratory or pulmonary function including spirometry, the arterial blood gas analysis, overnight pulse oximetry, peak cough expiratory flow, and diaphragmatic ultrasound [76,77], with the exception of forced vital capacity. Then, for some findings, the categorization resulted in very small sample sizes, such as for the respiratory phenotype. Finally, we did not take into account other possible confounders like drugs acting on glucose metabolism, that have recently been identified as potential factors in slowing on ALS progression [78,79]. 

## 5. Conclusions

This study examines the complex relationship between DM and ALS, revealing differential clinical features, prognostic factors, and therapeutic implications based on diabetes status. Our findings support the observation that respiratory impairment, but not a worse clinical status or a lower BMI, is associated with the presence of DM. To this regard, the literature data are still insufficient, requiring further studies to better understand the role of T2DM in respiratory function. Further, the underrepresentation of *C9ORF72* expansion in our diALS may hint towards a different impact in the interplay between metabolism and inflammation, warranting further studies for confirmation. The findings of this study offer valuable evidence that could deepen our understanding of the connection between T2DM and ALS. Therefore, investigating the molecular mechanisms underlying T2DM’s protective effect on ALS should be a key focus for ALS researchers. This exploration holds considerable therapeutic promise and could accelerate the development of much-needed treatments for ALS.

## Figures and Tables

**Table 1:** Clinical and demographic features of the patients stratified by the presence or absence of T2DM.

Variable	ndALS (*n* = 1611), *n* (%), Mean [SD]	diALS (*n* = 145), *n* (%), Mean [SD]	*p*-Value
Sex, male	878 (54.50)	96 (66.21)	0.007
Age at onset, y	65.76 [11.61]	71.56 [8.36]	<0.001
Age at diagnosis, y	66.82 [11.59]	72.89 [8.54]	<0.001
Diagnostic delay, m	13.81 [14.17]	15.93 [21.48]	0.101
BMI at diagnosis, kg/m^2^	24.23 [3.89]	25.63 [4.63]	<0.001
Weight change (%) *	−5.44 [8.12]	−6.87 [7.05]	0.070
FVC (%)	87.88 [24.21]	74.87 [26.48]	<0.001
Progression rate (from onset to diagnosis), points/month	0.96 [1.24]	1.23 [1.39]	0.022
Time to PEG, m	27.13 [18.46]	25.28 [18.22]	0.567
Time to NIV, m	28.03 [23.70]	23.66 [21.01]	0.141
Time to IV, m	31.25 [23.31]	25.26 [18.97]	0.224
ALSFRS-R at diagnosis	38.85 [7.17]	36.70 [7.63]	0.001
Site of onset			
Bulbar	535 (33.21)	48 (33.10)	0.979
Respiratory	42 (2.61)	9 (6.21)	0.013
UULL distal	428 (26.57)	24 (16.55)	0.008
UULL proximal	205 (12.73)	11 (7.59)	0.071
LLLL distal	496 (30.79)	52 (35.86)	0.207
LLLL proximal	291 (18.06)	31 (21.38)	0.323
Death **	914 (57.77)	93 (65.03)	0.092

Legend: diALS= ALS patients with a history of T2DM at diagnosis; ndALS = ALS patients without T2DM at diagnosis. BMI = Body Mass Index, PEG = Percutaneous Endoscopic Gastrostomy, NIV = Non-Invasive Ventilation, IV = Invasive Ventilation, ALSFRS-R = ALS Functional Rating Scale-Revised, UMNp = Upper Motor Neuron predominant phenotype, SD = Standard Deviation, UL = Upper Limb, LL = Lower Limb, UULL = both Upper Limbs, LLLL= both Lower Limbs. * Weight gain % has been defined as the percentage increase in weight at diagnosis, with respect to healthy body weight. A negative weight change (e.g., −5%), means a weight loss (e.g., of 5%). ** Death data were available for 1582 ndALS and for 143 diALS.

**Table 2:** Symptoms at onset of ALS patients stratified by diabetic status.

Type of ALS Onset	ndALS (*n* = 1611), *n* (%)	diALS (*n* = 145), *n* (%)	*p*-Value
Fasciculations	289 (17.94)	17 (11.72)	0.059
Cramps	235 (14.59)	13 (8.97)	0.063
Motor deficit	1411 (87.59)	138 (95.17)	0.007
Spasticity	102 (6.33)	3 (2.07)	0.038

Legend: diALS= ALS patients with a history of T2DM at diagnosis; ndALS = ALS patients without T2DM at diagnosis.

**Table 3:** (**a**) Genetics of the patients included in the study according to T2DM. (**b**) Clinical phenotypes of the patients included in the study according to T2DM.

(a)			
Genes *	ndALS (*n* = 1611), *n* (%)	diALS (*n* = 145), *n* (%)	*p*-Value
Genes			
*C9ORF72*	46 (7.64)	0	0.039
*SOD1*	24 (3.85)	2 (3.92)	0.980
*FUS*	8 (1.30)	1 (2.00)	0.678
*TARDBP*	8 (1.29)	0	0.423
*OPTN*	1 (0.16)	0	0.773
*FIG4*	6 (0.94)	0	0.479
*CHMP2B*	2 (0.31)	0	0.683
*VAPB*	1 (0.16)	0	0.773
*DCTN1*	3 (0.47)	0	0.617
*KIF5A*	3 (0.47)	0	0.617
*MAPT*	4 (0.62)	0	0.564
*SQSTM1*	3 (0.47)	0	0.615
(**b**)			
Phenotypes **	**ndALS** **(*n* = 1611), *n* (%)**	**diALS** **(*n* = 145), *n* (%)**	***p*** **-Value**
Phenotypes			0.138
Bulbar	456 (34.89)	42 (35.59)	1.000
Classic	612 (46.82)	57 (48.31)	0.924
Flail Arm	72 (5.51)	4 (3.39)	0.328
Flail Leg	69 (5.28)	7 (5.93)	0.831
UMN-predominant	79 (6.04)	5 (4.24)	0.543
Respiratory	19 (1.45)	5 (4.24)	0.045

Legend (**a**): diALS = ALS patients with a history of T2DM at diagnosis; ndALS = ALS patients without T2DM at diagnosis. Notes: * C9ORF72 data were available for 602 ndALS and for 52 diALS; SOD1 data were available for 623 ndALS and for 51 diALS; FUS data were available for 617 ndALS and for 50 diALS; TARDBP data were available for 618 ndALS and for 49 diALS; OPTN, FIG4 and VAPB data were available for 639 ndALS and for 53 diALS; DCTN1, KIF5A, and MAPT data were available for 640 ndALS and for 53 diALS; SQSTM1 data were available for 644 ndALS and for 54 diALS. Legend (**b**): diALS= ALS patients with a history of T2DM at diagnosis; ndALS = ALS patients without T2DM at diagnosis, UMN = Upper Motor Neuron. Notes: ** Phenotype classification was available for 1307 ndALS and for 118 diALS.

**Table 4:** Genetics and clinical features of the patients included in the study according to age classes and T2DM.

		Age Classes					
		<55 Years	55–64 Years	65–74 Years	75–84 Years	≥85 Years	*p*-Value
*C9ORF72* Expansion, *n* (%)	diALS	0 (0)	0 (0)	0 (0)	0 (0)	0 (0)	-
	ndALS	15 (32.61)	20 (43.48)	11 (23.91)	0 (0)	0 (0)	0.010
Respiratory onset, *n* (%)	diALS	1 (11.11)	1 (11.11)	4 (44.44)	3 (33.33)	0 (0)	0.773
	ndALS	1 (2.38)	9 (21.43)	21 (50.00)	10 (23.81)	1 (2.38)	0.086
FVC (%), mean [SD]	diALS	96.77 [27.92]	69.20 [18.89]	79.28 [27.17]	71.61 [27.77]	55.00 [7.07]	0.537
	ndALS	94.00 [22.44]	91.98 [22.84]	86.06 [23.54]	80.17 [26.33]	77.32 [26.44]	0.329
Progression rate, mean [SD]	diALS	1.64 [0.89]	0.88 [0.92]	1.21 [1.25]	1.33 [1.77]	1.18 [0.58]	0.002
	ndALS	0.75 [0.82]	0.74 [0.85]	0.94 [1.15]	1.38 [1.81]	1.28 [1.16]	<0.001
Total, *n* (%)	diALS	6 (4.14)	17 (11.72)	59 (40.69)	54 (37.24)	9 (6.21)	<0.001
	ndALS	260 (16.18)	382 (23.77)	563 (35.03)	348 (21.66)	54 (3.36)	

Legend: diALS= ALS patients with a history of T2DM at diagnosis; ndALS = ALS patients without T2DM at diagnosis. FVC = Forced Vital Capacity. SD = Standard Deviation.

**Table 5:** Comorbidities for ALS patients included in the study according to presence/absence of T2DM.

Comorbidities	ndALS (*n* = 1611), *n* (%)	diALS (*n* = 145), *n* (%)	*p*-Value
Frontotemporal dementia	151 (9.37)	11 (7.59)	0.476
Alzheimer’s disease	1 (0.06)	0 (0.00)	0.764
Vascular dementia	1 (0.06)	1 (0.69)	0.032
Other dementias	4 (0.25)	1 (0.69)	0.339
Parkinson’s disease	29 (1.80)	7 (4.83)	0.014
Bradykinesia	23 (1.43)	5 (3.45)	0.063
Tremor	23 (1.43)	1 (0.69)	0.463
Rigidity	21 (1.30)	3 (2.07)	0.447
Respiratory diseases	192 (11.92)	22 (15.17)	0.379
COPD	116 (7.20)	18 (12.41)	0.024
Thyroid disorder	174 (10.80)	21 (14.48)	0.419
Autoimmune diseases	116 (7.20)	4 (2.76)	0.537
Cardiovascular diseases	249 (15.46)	50 (30.48)	<0.001
Atrial fibrillation	74 (4.59)	9 (6.21)	0.381
Heart conduction disorders	27 (1.68)	4 (2.76)	0.343
Coronary artery disease	41 (2.55)	16 (11.03)	<0.001
Myocardial infarction	23 (1.43)	6 (4.14)	0.014
Hypertensive heart disease	30 (1.86)	10 (6.90)	<0.001
Valvular heart disease	20 (1.24)	2 (1.38)	0.886
Congestive heart failure	2 (0.12)	1 (0.69)	0.114
Other diseases	31 (1.92)	2 (1.38)	0.643
Hypertension	673 (41.78)	103 (71.03)	<0.001
Dyslipidemia	133 (8.04)	12 (11.76)	0.184
Neoplasms	293 (18.20)	39 (26.90)	0.337
Psychiatric diseases	132 (8.19)	8 (5.52)	0.254
Gastrointestinal diseases	228 (14.15)	18 (12.41)	0.563

Legend: diALS = ALS patients with a history of T2DM at diagnosis; ndALS = ALS patients without T2DM at diagnosis COPD = Chronic Obstructive Pulmonary Disease.

**Table 6:** Multivariable regression analysis of factors related to progression rate (points/month) at diagnosis.

Independent Variables	Regression Analysis	
	β (95% CI)	*p*-Value
Diabetes mellitus	0.45 (0.14–0.75)	0.004
Age at onset, years	0.01 (0.00–0.02)	0.007
Weight loss at diagnosis, kg	0.03 (0.02–0.04)	<0.001
*C9ORF72* expansion	0.42 (0.12–0.73)	0.006

Legend: BMI = Body Mass Index, COPD = Chronic Obstructive Pulmonary Disease, 95% CI = Confidence Interval.

**Table 7:** Logistic regression analyses of factors related to NIV treatment in our population.

Independent Variables	Regression Analysis	
	OR (95% CI)	*p*-Value
Diabetes mellitus	1.72 (0.98–3.02)	0.057
Age at onset, years	0.98 (0.97–1.00)	0.013
Respiratory onset	5.1 (1.67–15.58)	0.004
BMI at diagnosis, kg/m^2^	1.04 (1.00–1.08)	0.041
FVC	0.99 (0.98–1.00)	0.007

Legend: BMI = Body Mass Index, COPD = Chronic Obstructive Pulmonary Disease, OR = Odds Ratio, 95% CI = Confidence Interval.

**Table 8:** Multivariable Cox regression analysis of tracheostomy-free survival in ALS patients of the study.

Independent Variables	Multivariable Cox Regression Analysis	
	HR (95% CI)	*p*-Value
Age of onset, years	1.02 (1.00–1.03)	<0.001
Definite ALS according to EEC-R criteria	1.22 (1.03–1.44)	0.019
Possible ALS according to EEC-R criteria	0.78 (0.64–0.95)	0.018
Diagnostic delay, months	0.96 (0.95–0.97)	<0.001
Presence of FTD	1.38 (1.11–1.73)	0.003
Progression rate from onset to diagnosis points/month	1.19 (1.10–1.29)	<0.001
Weight loss, kg	1.02 (1.01–1.03)	<0.001
ALSFRS-R total score at diagnosis	0.98 (0.96–0.99)	0.013

Legend: EEC-R= El Escorial Criteria revised, FTD = Fronto-Temporal Dementia, ALSFRS-R = ALS Functional Rating Scale-Revised, HR = Hazard Ratio, 95% CI = Confidence Interval.

**Table 9:** Multivariable Cox regression analysis of tracheostomy-free survival in diALS.

Independent Variables	Multivariable Cox Regression Analysis	
	HR (95% CI)	*p*-Value
Definite ALS according to EEC-R criteria EC-R criteria	2.24 (1.38–3.83)	0.023
Diagnostic delay, m	0.97 (0.95–0.99)	0.013
Presence of FTD	2.24 (1.05–4.78)	0.037
Progression rate (from onset to diagnosis)	1.23 (1.03–1.48)	0.024
BMI, kg/m^2^	0.94 (0.89–0.99)	0.026
Respiratory onset	2.53 (1.07–5.99)	0.034

Legend: BMI = Body Mass Index, EEC-R= El Escorial Criteria revised, FTD = Fronto-Temporal Dementia, HR = Hazard Ratio, 95% CI = Confidence Interval.

**Table 10:** Multivariable Cox regression analysis of time to NIV in ALS patients of the study.

Variables for Time to NIV	Multivariable Cox Regression Analysis	
	HR (95% CI)	*p*-Value
Diagnostic delay, months	0.9 (0.93–0.96)	<0.001
Respiratory onset	2.89 (1.46–5.63)	0.002
Hypertension	1.37 (1.04–1.79)	0.023
Cardiovascular diseases	1.52 (1.09–2.13)	0.014
Progression rate (from onset to diagnosis)	2.10 (1.77–2.49)	<0.001
Weight loss, kg	1.05 (1.03–1.08)	<0.001
FVC	0.99 (0.98–0.99)	<0.001
Phenotypes (bulbar as reference)	0.81 (0.72–0.93)	0.002
T2DM	1.71 (1.07–2.74)	0.024

Legend: FVC = Forced Vital Capacity, T2DM = Type 2 Diabetes Mellitus; HR = Hazard Ratio, 95% CI = Confidence Interval.

**Table 11:** Multivariable Cox regression analysis of time to IV in ALS patients of the study.

Variables for Time to IV	Multivariable Cox Regression Analysis	
	HR (95% CI)	*p*-Value
Age at onset	1.07 (1.05–1.10)	<0.001
Diagnostic delay	0.94 (0.91–0.97)	<0.001
Progression rate (from onset to diagnosis)	1.45 (1.20–1.75)	<0.001
Phenotype (bulbar as reference)	0.83 (0.71–0.96)	0.012
*C9ORF72* expansion	2.66 (1.43–4.92)	0.002
Cardiovascular diseases	2.74 (1.49–5.03)	0.001

Legend: BMI = Body Mass Index, EEC-R = El Escorial Criteria revised, HR = Hazard Ratio, 95% CI = Confidence Interval.

## Data Availability

Data are available from the authors upon reasonable request and after providing the approval of the ethical committee.

## Supplementary Materials

The following supporting information can be downloaded at: https://www.mdpi.com/article/10.3390/life15060936/s1.

## Appendix A. List of Members of ERRALS Group

Project coordinator: Prof. J. Mandrioli
Collaborating centers:
Department of Neurosciences, Azienda Ospedaliero Universitaria di Modena and Department of Biomedical, Metabolic and Neural Sciences, University of Modena and Reggio Emilia, Modena, Italy (Jessica Mandrioli, Nicola Fini, Ilaria Martinelli, Elisabetta Zucchi, Giulia Gianferrari, Cecilia Simonini, Annalisa Gessani, Andrea Ghezzi, and Marco Vinceti);
Dipartimento di Scienze Biomediche e Neuromotorie, University of Bologna, and IRCCS Istituto delle Scienze Neurologiche di Bologna, Bellaria Hospital, Bologna, Italy (Veria Vacchiano, Luigi Bonan, Silvia De Pasqua and Rocco Liguori);
IRCCS Istituto delle Scienze Neurologiche di Bologna, Department of Neurology and Stroke Center, Maggiore Hospital, Bologna, Italy (Anna Maria Borghi and Andrea Zini);
IRCCS Istituto delle Scienze Neurologiche di Bologna, UOC Interaziendale Clinica Neurologica Metropolitana (NeuroMet), Bologna, Italy (Rita Rinaldi and Maria Guarino);
Department of Neurosciences and Rehabilitation, UOC Neurologia Provinciale, St Anna Hospital, Ferrara, Italy (Elisabetta Sette, Riccardo De Gennaro and Daniela Gragnaniello);
Department of Neuroscience and Rehabilitation, University of Ferrara, Ferrara, Italy (Maura Pugliatti);
Department of Neurology, IRCCS Arcispedale Santa Maria Nuova, Reggio Emilia, Italy (Elena Canali, Luca Codeluppi, and Franco Valzania);
Department of General and Specialized Medicine, University Hospital of Parma, Parma, Italy (Lucia Zinno, Filippo Stragliati, Pietro Anceschi, Andi Nuredini, Sonia Romano, Alessandro D’Orsi, Giorgia Libelli);
Department of Neurology, Fidenza Hospital, Parma, Italy (Doriana Medici and Giovanna Pilurzi);
Department of Neurology, G. Da Saliceto Hospital, Piacenza, Italy (Emilio Terlizzi and Paolo Immovilli);
Department of Neurology, Carpi Hospital, Modena, Italy (Mario Santangelo);
Department of Neurology, Imola Hospital, Bologna, Italy (Patrizia De Massis);
Department of Neurology, Faenza and Ravenna Hospital, Ravenna, Italy (Matteo Gizzi, Marco Currò Dossi, Pietro Querzani and Maria Grazia Piscaglia);
Department of Neurology, Bufalini Hospital, Cesena, Italy (Simonetta Morresi, Maria Vitiello, and Marco Longoni);
Department of Neurology, Forlì Hospital, Forlì, Italy (Alberto Patuelli, Susanna Malagù, Francesca Bianchi, and Marco Longoni);
Department of Neurology, Infermi Hospital, Rimini, Italy (Cristiana Ganino, Tommaso Baldini and Claudio Callegarini);
Department of Hospital Services, Emilia Romagna Regional Health Authority, Bologna, Italy (Salvatore Ferro).
