# Supplementary material for: Exploring the Role of Diabetes in ALS: A Population-Based Cohort Study
Source: Life (Basel). 2025 Jun 10;15(6):936. doi: 10.3390/life15060936 (PMC12194302; doi:10.3390/life15060936)
Supplement: Supplementary file 1 [file life-15-00936-s001.zip › life-3673194-supplementary.pdf]

SUPPLEMENTARY TABLES for paper "Exploring the role of diabetes in ALS: a population-based study"

Table S1: Family history for neurodegenerative diseases of the ALS patients included in the study stratified by the presence or absence of diabetes mellitus.

Family history for neuro-degenerative diseases	ndALS (n=1611), n (%)	diALS(n=145), n (%)	p-value
**ALS**	92 (5.71)	3 (2.07)	0.063
**Dementia**	187 (11.61)	10 (6.90)	0.085
**Parkinson's Disease**	75 (4.66)	8 (5.52)	0.640

Legend: diALS: ALS patients with diabetes mellitus; ndALS: ALS patients without diabetes.

Table S2: Distribution of patients according to Revised El Escorial diagnostic criteria at diagnosis

Revised El Escorial Criteria	ndALS (n=1611), n (%)	diALS(n=145), n (%)	p-value
**Definite**	407 (25.26)	38 (26.21)	0.803
**Clinically Probable**	497 (30.85)	47 (32.41)	0.697
**Probable laboratory supported**	206 (12.79)	20 (13.79)	0.729
**Possible**	298 (18.50)	25 (17.24)	0.708

Legend: diALS: ALS patients with diabetes mellitus; ndALS: ALS patients without diabetes.

Table S3: Medications taken by the ALS patients included in the study stratified by the presence or absence of diabetes mellitus.

Medications	ndALS (n=1611), n (%)	diALS(n=145), n (%)	p-value
**Riluzole**	1,337 (82.99)	123 (84.83)	0.572
**Vitamine E**	835 (51.83)	68 (46.90)	0.255
**Vitamine C**	276 (17.13)	27 (18.62)	0.650
**Coenzyme Q**	19 (1.18)	0 (0.00)	0.189
**Acetyl-L-carnitine**	466 (28.93)	25 (17.24)	**0.003**
**Tauroursodeoxycholic acid**	73 (4.53)	3 (2.07)	0.163

Legend: diALS: ALS patients with diabetes mellitus; ndALS: ALS patients without diabetes.

Table S4: Non-pharmacological treatments of patients in the study based on diabetes mellitus status

Treatments*	ndALS (n=1611), n (%)	diALS(n=145), n (%)	p-value
**PEG**	499 (31.56)	35 (24.48)	0.079
**NIV**	595 (37.61)	72 (50.35)	**0.003**
**IV**	261 (16.50)	24 (16.78)	0.930

Legend: diALS: ALS patients with diabetes mellitus, PEG=Percutaneous Endoscopic Gastrostomy, ndALS: ALDS patients without diabetes, NIV=Non-Invasive Ventilation, IV=Invasive Ventilation. **Notes**: *PEG data were available for 1581 patients among those without diabetes and for 143 among those with concomitant diabetes; NIV and IV data were available for 1582 patients among those without diabetes and for 143 among those with concomitant diabetes.

Table S5: Univariate Cox regression analysis of tracheostomy-free survival in ALS patients of the study.

Variables	HR	95% CI	p-value
**Sex, male**	0.97	0.86-1.09	0.681
**Age of onset, years**	1.02	1.01-1.02	**<0.001**
**Diagnostic delay, months**	0.96	0.95-0.97	**<0.001**
**Family history for ALS**	0.76	0.57-1.01	0.065
**Type of onset**			
Spinal (reference)	1		
Bulbar	1.41	1.24-1.60	**<0.001**
**Phenotypes**	0.86	0.81-0.92	**<0.001**
Bulbar (reference)	1		
Classic	0.72	0.62-0.83	**<0.001**
Flail Arm	0.66	0.50-0.90	**0.007**
Flail Leg	0.57	0.41-0.78	**0.001**
UMN-predominant	0.43	0.31-0.60	**<0.001**
Respiratory	1.41	0.91-2.17	0.116
**Onset with fasciculations**	1.12	0.96-1.31	0.120
**Onset with cramps**	0.83	0.70-0.99	**0.045**
**Onset with motor deficit**	1.35	1.11-1.65	**0.003**
**Onset with spasticity**	0.75	0.58-0.97	**0.033**
**Time to generalization, months**	0.95	0.93-0.96	**<0.001**
**ALSFRS-R total score, points**	0.97	0.96-0.98	**<0.001**
**Progression rate at diagnosis, points/month**	1.17	1.14-1.20	**<0.001**
**Weight loss at diagnosis, kg**	1.03	1.02-1.04	**<0.001**
**BMI, kg/m²**	0.98	0.96-1.00	**0.042**
**FVC, %**	0.99	0.98-0.99	**<0.001**
**R-EEC Definite**	1.71	1.50-1.96	**<0.001**
**R-EEC clinically probable**	1.04	0.92-1.18	0.523
**R-EEC probable laboratory supported**	0.83	0.69-1.00	**0.047**
**R-EEC possible**	0.70	0.59-0.82	**<0.001**
**FTD**	1.50	1.24-1.82	**<0.001**
**Comorbidities**			
Diabete mellitus	1.17	0.95-1.44	0.138
Parkinsonism	1.38	0.94-2.02	0.098
Dyslipidemia	0.91	0.68-1.21	0.533
Hypertension	1.26	1.11-1.42	**<0.001**

COPD	1.20	0.96-1.48	0.096
Other Respiratory Diseases	0.75	0.62-0.92	**0.005**
Dysthyroidism	0.79	0.64-0.97	**0.025**
Cardiovascular Diseases	1.03	1.08-1.48	**0.003**
Autoimmune Diseases	0.88	0.68-1.12	0.298
Neoplasm	0.84	0.71-0.98	**0.028**
Psychiatric Diseases	1.05	0.84-1.32	0.622
Gastrointestinal Diseases	1.00	0.84-1.19	0.973

Legend: ALS= Amyotrophic Lateral Sclerosis, ALSFRS-R=ALS Functional Rating Scale-Revised, BMI=Body Mass Index, 95% CI=Confidence Interval, COPD=Chronic Obstructive Pulmonary Disease, FTD=Fronto-Temporal Dementia, FVC = Forced Vital Capacity, HR=Hazard Ratio, R-EEC= Revised El Escorial Criteria, UMN=Upper Motor Neuron.

Table S6. Multivariate Cox regression analysis of tracheostomy-free survival in ALS patients of the study without diabetes.

**Variables for tracheostomy-free survival**	**Multivariate Cox regression analysis**	
	**HR (95% CI)**	**p-value**
**Diagnostic delay, m**	0.97 (0.96-0.98)	**<0.001**
**Age at onset**	1.01 (1.00-1.02)	**<0.001**
**Bulbar onset**	1.20 (1.03-1.41)	**0.019**
**Weight loss, kg**	1.02 (1.01-1.04)	**<0.001**
**Progression rate (from onset to diagnosis)**	1.30 (1.22-1.39)	**<0.001**
**FTD**	1.39 (1.09-1.74)	**0.007**

Legend: FTD=Fronto-Temporal Dementia, HR=Hazard Ratio, 95% CI=Confidence Interval

Table S7: Univariate Cox regression analysis of tracheostomy-free survival in ALS patients of the study diagnosed with diabetes mellitus.

Variable	HR	95% CI	p-value
**Sex, male**	1.14	0.74-1.74	0.543
**Age of onset, years**	0.99	0.97-1.01	0.707
**Diagnostic delay, months**	0.97	0.95-0.98	**0.001**
**Family history for ALS**	0.47	0.07-3.40	**0.450**
**Type of onset**			
Bulbar	0.83	0.53-1.30	0.431
Respiratory	4.45	2.10-9.43	**<0.001**
**Phenotypes**	1.06	0.89-1.28	0.469
Bulbar (reference)	1		
Classic	0.96	0.58-1.58	0.895
Flail arm	1.09	0.38-3.14	0.868
Flail leg	0.75	0.26-2.16	0.601
UMN-predominant	0.59	0.14-2.51	0.480
Respiratory	3.28	1.23-8.74	**0.017**
**Onset with Fasciculations**	1.18	0.65-2.12	0.576
**Onset with Cramps**	0.74	0.36-1.54	0.435
**Onset with motor deficit**	1.19	0.43-3.26	0.725
**Onset with spasticity**	0.64	0.15-2.60	0.534
**Time of generalization, months**	0.97	0.95-1.00	0.117
**ALSFRS-R total score, points**	0.95	0.92-0.98	**0.002**
**Progression rate at diagnosis, points/month**	1.43	1.25-1.63	**<0.001**
**Weight loss at diagnosis, kg**	1.04	1.00-1.09	**0.038**
**BMI, kg/m^2**	0.94	0.89-0.98	**0.018**
**FVC, %**	0.98	0.97-0.99	**0.026**
**R-EEC Definite**	2.22	1.44-3.42	**<0.001**
**R-EEC clinically probable**	0.96	0.62-1.47	0.871
**R-EEC probable laboratory supported**	0.61	0.33-1.13	0.121
**R-EEC possible**	0.87	0.50-1.48	0.613
**FTD**	2.38	1.22-4.63	**0.011**
**Comorbidities**			
Parkinsonism	1.18	0.48-2.91	0.712
Dyslipidemia	1.48	0.74-2.94	0.263
Hypertension	1.51	0.94-2.42	0.083
COPD	1.38	0.79-2.40	0.248
Other Respiratory Diseases	1.39	0.81-2.37	0.234

Dysthyroidism	1.18	0.67-2.09	0.551
Cardiovascular Diseases	1.40	0.93-2.10	0.106
Autoimmune Diseases	1.02	0.32-3.23	0.970
Neoplasm	1.10	0.70-1.73	0.668
Psychiatric Diseases	1.61	0.32-3.23	0.254
Gastrointestinal Diseases	0.88	0.48-1.61	0.690

Legend: ALS= Amyotrophic Lateral Sclerosis, ALSFRS-R=ALS Functional Rating Scale-Revised, BMI=Body Mass Index, 95% CI=Confidence Interval, COPD=Chronic Obstructive Pulmonary Disease,FTD=Fronto-Temporal Dementia, FVC = Forced Vital Capacity, HR=Hazard Ratio, R-EEC= Revised El Escorial Criteria, UMN=Upper Motor Neuron.

Table S8: Univariate Cox regression analysis of time to NIV in ALS patients of the study

Variable	HR	95% CI	p-value
**Sex, male**	0.96	0.82 - 1.13	0.640
**Age of onset, years**	1.02	1.02 - 1.03	**<0.001**
**Diagnostic delay, months**	0.95	0.94 - 0.96	**<0.001**
**Family history for ALS**	0.92	0.67-1.27	0.609
**Type of onset**			
Bulbar	1.58	1.33 - 1.87	**<0.001**
Respiratory	4.62	3.21 - 6.68	**<0.001**
**Phenotypes**	0.89	0.82-0.97	**0.007**
Bulbar (reference)	1		
Classic	0.64	0.53-0.77	**<0.001**
Flail arm	0.53	0.36-0.78	**0.001**
Flail leg	0.53	0.33-0.84	**0.007**
UMN-predominant	0.41	0.26-0.64	**<0.001**
Respiratory	3.31	1.97-5.55	**<0.001**
**Onset with Fasciculations**	0.98	0.81-1.20	0.862
**Onset with Cramps**	0.74	0.59-0.91	**0.005**
**Onset with motor deficit**	1.09	0.83-1.43	0.527
**Onset with spasticity**	0.88	0.61-1.25	0.470
**Time of generalization, months**	0.88	0.72-1.08	0.217
**ALSFRS-R total score, points**	0.98	0.97 - 0.99	**0.001**
**Progression rate at diagnosis, points/month**	2.07	1.93 - 2.23	**<0.001**
**Weight loss at diagnosis, kg**	1.02	1.01-1.03	**0.001**
**BMI, kg/m^2**	0.98	0.96 - 1.00	0.212
**FVC, %**	0.99	0.99 - 0.99	**<0.001**
**R-EEC Definite**	1.41	1.18-1.68	**<0.001**
**R-EEC clinically probable**	0.90	0.76-1.06	0.219
**R-EEC probable laboratory supported**	0.97	0.77-1.22	0.785
**R-EEC possible**	0.79	0.65-0.97	**0.026**
**FTD**	1.06	0.76 - 1.49	0.698
**Comorbidities**			
Diabetes Mellitus	1.23	0.96 - 1.58	0.097
Parkinsonism	0.80	0.46-1.39	0.425
Dyslipidemia	0.92	0.67 - 1.26	0.619
Hypertension	1.26	1.08 - 1.48	**0.003**
COPD	1.23	0.93 - 1.64	0.145
Other Respiratory Diseases	0.95	0.76-1.20	0.688

Dysthyroidism	0.98	0.78 - 1.25	0.922
Cardiovascular Diseases	1.31	1.07 - 1.60	**0.007**
Autoimmune Diseases	0.91	0.67-1.24	0.561
Neoplasm	1.22	1.00-1.50	0.052
Psychiatric Diseases	1.25	0.94 - 1.66	0.120
Gastrointestinal Diseases	0.99	0.80-1.23	0.913
**Genes mutations**			
C9ORF72	1.74	1.13 - 2.68	**0.011**
FUS	2.97	0.73 - 12.03	0.127
SOD1	0.78	0.42 - 1.43	0.425

Legend: ALS= Amyotrophic Lateral Sclerosis, ALSFRS-R=ALS Functional Rating Scale-Revised, BMI=Body Mass Index, 95% CI=Confidence Interval, COPD=Chronic Obstructive Pulmonary Disease,FTD=Fronto-Temporal Dementia, FVC = Forced Vital Capacity, HR=Hazard Ratio, R-EEC= Revised El Escorial Criteria, UMN=Upper Motor Neuron.

Table S9: Univariate Cox regression analysis of time to IV in ALS patients of the study

Variable	HR	95% CI	p-value
**Sex, male**	1.02	0.80 - 1.30	0.840
**Age of onset, years**	1.04	1.03 - 1.06	**<0.001**
**Diagnostic delay, months**	0.95	0.94 - 0.97	**<0.001**
**Family history for ALS**	1.46	0.84-2.56	0.183
**Type of onset**			
Bulbar	1.37	1.06 - 1.78	**0.015**
Respiratory	1.10	0.69 - 1.74	0.685
**Phenotypes**	0.91	0.82-1.01	0.087
Bulbar (reference)	1		
Classic	0.80	0.60-1.07	0.131
Flail arm	0.52	0.29-0.94	**0.031**
Flail leg	0.88	0.47-1.64	0.682
UMN-predominant	0.61	0.31-1.19	0.149
Respiratory	0.74	0.38-1.45	0.386
**Onset with Fasciculations**	0.98	0.74-1.31	0.905
**Onset with Cramps**	0.77	0.55-1.07	0.123
**Onset with motor deficit**	0.93	0.64-1.35	0.704
**Onset with spasticity**	0.83	0.48-1.43	0.504
**Time of generalization, months**	0.89	0.64-1.24	0.511
**ALSFRS-R total score, points**	0.97	0.96 - 0.98	**<0.001**
**Progression rate at diagnosis, points/month**	1.45	1.34 - 1.57	**<0.001**
**Weight loss at diagnosis, kg**	1.03	1.01-10.5	**0.005**
**BMI, kg/m^2**	0.96	0.93 - 0.99	**0.032**
**FVC, %**	0.99	0.99 - 1.00	0.151
**R-EEC Definite**	1.68	1.29-2.18	**<0.001**
**R-EEC clinically probable**	1.28	0.99-1.66	0.056
**R-EEC probable laboratory supported**	0.70	0.45-1.09	0.114
**R-EEC possible**	0.61	0.46-0.82	**0.001**
**FTD**	2.35	1.50 - 3.70	**<0.001**
**Comorbidities**			
Diabetes Mellitus	1.35	0.89 - 2.06	0.160
Parkinsonism	0.85	0.38-1.90	0.686
Dyslipidemia	1.11	0.64 - 1.90	0.780
Hypertension	1.38	1.08 - 1.76	**0.009**
COPD	1.25	0.83 - 1.89	0.287
Other Respiratory Diseases	1.06	0.69-1.65	0.786

Dysthyroidism	0.93	0.63 - 1.37	0.713
Cardiovascular Diseases	1.56	1.11 - 2.19	**0.011**
Autoimmune Diseases	1.32	0.70-2.49	0.388
Neoplasm	1.25	0.92-1.69	0.148
Psychiatric Diseases	1.47	0.97 - 2.18	0.068
Gastrointestinal Diseases	0.99	0.70-1.40	0.955
**Genes mutations**			
C9ORF72	1.89	1.06 - 3.39	**0.031**
FUS	1.65	0.23 - 11.96	0.618
SOD1	1.03	0.42 - 2.55	0.946

Legend: ALS= Amyotrophic Lateral Sclerosis, ALSFRS-R=ALS Functional Rating Scale-Revised, BMI=Body Mass Index, 95% CI=Confidence Interval, COPD=Chronic Obstructive Pulmonary Disease, FTD=Fronto-Temporal Dementia, FVC = Forced Vital Capacity, HR=Hazard Ratio, R-EEC= Revised El Escorial Criteria, UMN=Upper Motor Neuron.
